# Supplementary material for: Cardiovascular disease and subsequent risk of psychiatric disorders: a nationwide sibling-controlled study
Source: eLife. 2022 Oct 21;11:e80143. doi: 10.7554/eLife.80143 (PMC9718522; doi:10.7554/eLife.80143)
Supplement: Supplementary file 1. — (a) Summary of prospective cohort studies addressing the association between various indications of cardiovascular disease and risk of psychiatric disorders/psychiatric symptoms. (b) International Classification of Diseases (ICD) codes for exposure, outcome, and covariates identifications. (c) Crude incidence rates (IRs) and hazard ratios (HRs) with 95% confidence intervals (CIs) for incident psychiatric disorder among CVD patients compared with their full siblings or matched population controls, by patient characteristics. CVD: cardiovascular disease. aCox regression models, stratified by family identifier for sibling comparison or matching identifier (birth year and sex) for population comparison, adjusting for sex, birth year, educational level, individualized family income, cohabitation status, history of somatic disease, and family history of psychiatric disorder. Time since index date was used as underlying time scale. (d) Crude incidence rates (IRs) and hazard ratios (HRs) with 95% confidence intervals (CIs) for incident psychiatric disorders among CVD patients compared with their full siblings or matched population controls, by time of follow-up (<1 or ≥ 1 year from CVD diagnosis). CVD: cardiovascular disease. aCox regression models, stratified by family identifier for sibling comparison or matching identifier (birth year and sex) for population comparison. Time since index date was used as underlying time scale. (e) Crude incidence rates (IRs) of different types of psychiatric disorder among CVD patients, their full siblings, and matched population controls, by time of follow-up (<1 or ≥ 1 year from CVD diagnosis). CVD: cardiovascular disease. (f) Crude incidence rates (IRs) for psychiatric disorders among different groups of CVD patients, their full siblings and matched population controls, by time of follow-up (<1 or ≥ 1 year from CVD diagnosis)a. CVD: cardiovascular disease. aWe identified all cardiovascular diagnoses during follow-up and considered CVD [file elife-80143-supp1.docx]

**Supplementary file 1**

**1a. Summary of prospective cohort studies addressing the association between various indications of cardiovascular disease and risk of psychiatric disorders/psychiatric symptoms.**

| Paper | Study design and database | Exposure | Outcome | Sample size and follow-up | Main findings | Control for | |
| --- | --- | --- | --- | --- | --- | --- | --- |
|  |  |  |  |  |  | familial factors? | other cardiovascular comorbidities? |
| Petersson S, 2014^1^ | population-based cohort study; basedline measured between 2000 and 2006 | hypertansion and stroke in structured interviews | incident depressive disorders | 567 very old people≥85 years； with 5 years of follow-up; . | hypertension OR 2.83 (1.08-7.42) and history of stroke OR 3.25 (1.12-9.44) | no | no |
| Zhang X, 2015^2^ | Community-based study; random sampling between March 1999 and February 2001 | arrhythmia and heart failgure in self-report questionnaire | Generalized anxiety disorder (GAD) | 1711 individuals aged 65 years and above; median follow-up time was 9.7 years | arrhythmia and heart failure HR 1,67 (1.04-2.70) | no | no |
| Chang JC, 2017^3^ | longidtudinal cohort study; a commuity health-screening program between 1999-2004 in Taiwan | clinical diagnosis of cardiovascular disease | clinical diagnosis of incident posttraumatic stress disorder (PTSD) | 76,417 aged 30 years and above and mean follow-up of 3.13 (1-5) years | cardiovascular disease HR 1.45 (1.03–2.04) | no | no |
| Feng HP, 2016^4^ | cohort study; National Health Insurance Research Database in Taiwan during January 1 and December 31, 2005 | clinically diagnosed myocardial infarction | clinical diagnosis of anxiety or depressive disorder | 1396 patients with myocardial infarction and matched 13,960 individuals without MI (63% men); follow-up of 2 years | HR 5.06 (4.61–5.54) for anxiety disorders and HR 7.23 (4.88–10.88) for depressive disorders | no | control for hypertension and cerebrovascular disease in model |
| Kivimäki M, 2012^5^ | prospective cohort study; London-based office staff, self-reported questionnaire and medical records | Vascular risk assessed with the Framingham Cardiovascular, Coronary Heart Disease, and Stroke Risk Scores | New depressive symptoms identified by General Health Questionnaire (GHQ)>=5, and Center for Epidemiologic Studies Depression Scale score (CES-D)>=16, and use of antidepressant medication. | 5318 participants (mean age 54.8 years, 31% women); baseline examination in 1991 and follow-up screenings in1997, 2003, and 2008 | Diagnosed vascular disease (coronary heart disease or stroke) with an increased risk for depressive symptoms, OR 1.5 (1.0 –2.2) to 2.0 (1.4 –3.0) depending on indicators | no | no |
| Zimmerman JA, 2009^6^ | Logitudinal study among community dwelling elder Mexican Americans during 1993-1994 | self-reported vascular risk factors (hypertension, storke and myocardial infarction) | Depressive symptom  Measured with Center for Epidemiologic Studies Depression Scale (CES-D>=16) | 964 participants (mean age 72) with two year of follow-up; 53% women | Hypertension: OR 2.3 (1.39-3.70); stroke: OR 0.83 (0.25-2.74); myocardial infarction: 1.48 (0.71-3.10) | no | no |
| Kim JM, 2006^7^ | community-based prospective study from 2001-2003 | self-reported vascular disease risk (stroke, heart disease, hypertension) | Depression assessed using the community version of the Geriatric Mental State (GMS) | 661 community participants (mean age 73) with two year of follow-up; 55% women | Pre-existing heart disease: OR 2.2 (1.3-3.7); incident stroke: OR 2.2 (1.0-5.0) | no | no |
| Mast BT, 2008^8^ | Community residents | prevalent vascular disease | incidence depressive symptoms using Center for Epidemiologic Studies Depression (CES-D>8) scale. | 1,796 elders ages 70–79 with two year of follow-up; | Coronary heart disease: OR 1.68 (1.09-2.59); myocardial infarction: OR 2.69 (0.99-7.28); atrial fibrillation: OR 0.80 (0.19-3.42); left ventricular hypertrophy: OR 1.17 (0.65-2.12); congestive heart failure: OR 0.90 (0.27-3.06); hypertension: OR 1.07 (0.73-1.57); phripheral arterial disease: OR 1.58 (0.75-3.33) | no | Charlson index |
| Godin O, 2012^9^ | multicenter cohort study with random sample during 1999-2010 | Measures of systolic and diastolic blood pressure or using antihypertensive medication | major depressive symptoms according to the Mini-International Neuropsychiatric Interview or taking antidepressant medication | 3,090 with ten years of follow-up; 59.9% female | Hypertension: RR 0.97 (0.76-1.22) | no | no |
| Garcia-Fabela L, 2009^10^ | longitudinal study of community-dwelling persons | self-reported  hypertension | depressive symptoms | 3,276 persons aged 60 years and older with two year of follow-up; | Hypertension: OR 1.18 (1.01–1.40) | no | Adjust for stroke, ischemic cardiopathy in model |
| Simning A, 2018^11^ | nationally representative longitudinal survey of U.S. Medicare beneficiaries initiated in 2011 | new onset  heart attack or stroke in interview | PHQ-2 identified clinically significant depressive symptoms | 5,643 aged 65 years and older with one year follow-up | Among those no close contact: heart attack OR 5.57 (1.68-18.44), stroke OR 4.44 (1.03-19.14); among those with close contacts: heart attack OR 1.19 (0.68-2.07), stroke OR 2.51 (1.76-3.59) | no | Adjust for medical conditions (include hypertension and heart disease) |
| Chuang CS, 2014^12^ | Cohort study using National Health Insurance (NHI) data sets from Taiwan in period 2000-2002 | hyperlipidermia (comorbidity as hypertension) from insurrance database | an insurance claim for diagnosis and [treatment of depression](https://www.sciencedirect.com/topics/medicine-and-dentistry/treatment-of-depression) | 26,852 exposed and 107,408 unexposed individuals | Hypertension: HR 1.70 (1.57–1.83) | no | no |

^1^Petersson, S. et al (2014). “Risk factors for depressive disorders in very old age: a population-based cohort study with a 5-year follow-up.” Soc Psychiatry Psychiatr Epidemiol 49, 831–839

^2^ Zhang, X. et al (2015). “Risk factors for late-onset generalized anxiety disorder: results from a 12-year prospective cohort (The ESPRIT study).” Transl Psychiatry 5, e536

^3^ Chang, JC. et al (2017). “Comorbid diseases as risk factors for incident posttraumatic stress disorder (PTSD) in a large community cohort (KCIS no.PSY4).” Sci Rep 7, 41276.

^4^ Feng, H-P et al. “Risk of anxiety and depressive disorders in patients with myocardial infarction: A nationwide population-based cohort study.” Medicine vol. 95,34 (2016): e4464.

^5^ Kivimäki M et al (2012). “Vascular risk status as a predictor of later-life depressive symptoms: a cohort study.” Biological Psychiatry;72:324 –330

^6^ Zimmerman JA et al (2009). “Vascular risk and depression in the Hispanic Established Population for the Epidemiologic Study of the Elderly (EPESE).” Int J Geriatr Psychiatry; 24: 409–416.

^7^ Kim JM et al (2006). “Vascular risk factors and incident late-life depression in a Korean population.” Br J Psychiatry; 189: 26–30.

^8^ Mast BT et al (2008). “Vascular disease and future risk of depressive symptomatology in older adults: findings from the Health, Aging, and Body Composition study.” Biol Psychiatry; 64: 320–326.

^9^ Godin O et al (2012). “Body mass index, blood pressure, and risk of depression in the elderly: a marginal structural model.” Am J Epidemiol; 176: 204–213.

^10^ Garcia-Fabela L et al (2009). “Hypertension as a risk factor for developing depressive symptoms among community-dwelling elders.” Rev Invest Clin; 61: 274–280.

^11^ Simning A et al (2018). “The association of a heart attack or stroke with depressive symptoms stratified by the presence of a close social contact: findings from the National Health and Aging Trends Study Cohort.” International journal of geriatric psychiatry vol. 33,1: 96-103.

^12^ Chuang CS et al (2014). “Hyperlipidemia, statin use and the risk of developing depression: a nationwide retrospective cohort study.” Gen Hosp Psychiatry;36(5):497-501.

**1b. International Classification of Diseases (ICD) codes for exposure, outcome and covariates identifications.**

|  | ICD-9 | ICD-10 |
| --- | --- | --- |
| Any psychiatric disorder | 291,292, 295-311 | F10-F69 |
| Non-affective psychotic disorders | 295, 297, 298, excluding 295H and 298B | F20-24, F28-29 |
| Affective psychotic disorders | 296, 295H, 298B | F25, F30-31, F32.3, F33.3 |
| Alcohol or drug misuse | 291, 292, 303, 304 | F10-16, F18-19 |
| Mood disorders, excluding those with psychotic symptoms | 300E, 311 | F32-34, F38-39, excluding F32.3 and F33.3 |
| Anxiety and stress related disorders | 300A-D, 300F-H, 300W-X, 306, 307A, 308, 309 | F40-48 |
| -Acute stress reaction | 308, 309A | F43.0 |
| Eating disorders | 307B, 307F | F50 |
| Personality disorders | 301 | F60-63, F68-69 |
|  |  |  |
| Any cardiovascular disease | 390-438, 440,444,445 | I00-I70, I730, I74- I75 |
| Ischemic heart disease | 410-414 | I20-I24, I25 (excluding I25.5) |
| Cerebrovascular disease | 430-434, 436-438 | I60-I69 |
| Emboli and thrombosis | 415, 444,445 | I26, I74, I75 |
| Hypertensive disease | 401-405 | I10-I16, I674 |
| Heart failure | 428 | I25.5, I42.0, I42.8, I42.9, I50 |
| Arrhythmia/conduction disorder | 426, 427 | I44-I49 |
|  |  |  |
| Covariates: history of severe somatic conditions |  |  |
| Chronic pulmonary disease | 500-505, 506C | J60-J67, J684, J70, J703 |
| Connective tissue disease | 710A, 710B, 710E, 714A, 714B, 714C, 714W,714X, 725 | M05, M06, M315, M32-M34, M351, M353, M360 |
| Diabetes | 250A, 250G | E100, E101, E106, E108, E109, E110, E111, E116, E118, E119, E120, E121, E126, E128, E129, E130, E131, E136, E138, E139, E140, E141, E146, E148, E149 |
| Diabetes with end organ damage | 250D-250F | E102-E105, E107, E112-E115, E117, E122-E125, E127, E132-E135, E137, E142-E145, E147 |
| Moderate or severe renal disease | 582, 583A, 583B, 583C, 583E, | I120, I131, N032-N037, N052-N057, N18, N19, N250, Z490-Z492, Z940, Z992 |
| Mild liver disease | 583G, 583H, 585, 586, 588 | B18, K700-K703, K709, K713-K715, K717, K73, K74, K760, K762--K764, K768, K769, Z944 |
| Moderate or severe liver disease | 571C, 571E,571F, 571G | I850, I859, I864, I982, K704, K711, K721, K729, K765, K766, K767 |
| Ulcer disease | 572C, 572D, 572E, 572W, 456A, 456B, 456C | K25-K28 |
| Any malignancy, including leukemia and lymphoma | 140-172, 174-195, 200-208 | C00-C26, C30-C34, C37-C41, C43, C45-C58, C60-C76, C81-C85, C88, C90-C97 |
| Metastatic cancer | 196,197,198,199A, 199B | C77- C80 |
| HIV/AIDS | 042, 043, 044 | B20-B22, B24 |
|  |  |  |

**1c. Crude incidence rates (IRs) and hazard ratios (HRs) with 95% confidence intervals (CIs) for incident psychiatric disorder among CVD patients compared with their full siblings or matched population controls, by patient characteristics**

|  | Sibling comparison | | | | | Population comparison | | | | |
| --- | --- | --- | --- | --- | --- | --- | --- | --- | --- | --- |
|  | Unaffected full siblings | | CVD patients | | | Matched population controls | | CVD patients | | |
|  | No. of cases | IR (per 1000 person years) | No. of cases | IR (per 1000 person years) | HR (95% CI)^a^ | No. of cases | IR (per 1000 person years) | No. of cases | IR (per 1000 person years) | HR (95% CI)^a^ |
| **Overall** | 38 367 | 4.6 | 25 793 | 7.0 | 1.55 (1.53-1.58) | 289 437 | 4.0 | 41 479 | 7.1 | 1.67 (1.65-1.69) |
| **By sex** |  |  |  |  |  |  |  |  |  |  |
| male | 17 368 | 4.4 | 14 196 | 6.4 | 1.51 (1.46-1.56) | 147 978 | 3.4 | 22 546 | 6.4 | 1.80 (1.78-1.83) |
| female | 20 999 | 4.8 | 11 597 | 8.0 | 1.56 (1.50-1.62) | 141 459 | 4.9 | 18 933 | 8.0 | 1.58 (1.55-1.60) |
| **By age at cohort entry** |  |  |  |  |  |  |  |  |  |  |
| <50 | 21 391 | 5.9 | 13 324 | 9.4 | 1.51 (1.47-1.55) | 158 883 | 5.7 | 22 046 | 9.4 | 1.54 (1.52-1.57) |
| 50-60 | 11 316 | 3.8 | 7 598 | 5.9 | 1.55 (1.48-1.62) | 81 273 | 2.9 | 12 682 | 5.6 | 1.95 (1.91-1.99) |
| >60 | 5 660 | 3.2 | 4 871 | 5.1 | 1.69 (1.60-1.78) | 49 281 | 3.1 | 6 751 | 5.3 | 1.74 (1.70-1.79) |
| **By age during follow-up** |  |  |  |  |  |  |  |  |  |  |
| <60 | 25,165 | 10.2 | 16 995 | 15.0 | 1.51 (1.47-1.56) | 169 074 | 10.5 | 24 592 | 16.4 | 1.48 (1.46-1.51) |
| >=60 | 13,202 | 2.3 | 8 798 | 3.5 | 1.46 (1.41-1.51) | 120 363 | 2.1 | 16 887 | 3.9 | 1.77 (1.74-1.80) |
| **By calendar year at cohort entry** |  |  |  |  |  |  |  |  |  |  |
| 1987-1996 | 9 288 | 3.7 | 4 931 | 5.1 | 1.49 (1.43-1.55) | 66 521 | 3.5 | 7 648 | 5.1 | 1.43 (1.39-1.46) |
| 1997-2006 | 18 905 | 5.0 | 12 144 | 7.2 | 1.49 (1.45-1.54) | 141 660 | 4.4 | 18 996 | 7.2 | 1.58 (1.55-1.60) |
| 2007-2016 | 10 174 | 5.0 | 8 718 | 8.4 | 1.69 (1.63-1.74) | 81 256 | 4.0 | 14 835 | 8.6 | 2.01 (1.97-2.04) |
| **By history of somatic disease** |  |  |  |  |  |  |  |  |  |  |
| No | 35 983 | 4.6 | 23 223 | 6.9 | 1.56 (1.52-1.59) | 265 039 | 3.9 | 36 847 | 6.9 | 1.71 (1.69-1.73) |
| Yes | 2 384 | 5.2 | 2 570 | 8.2 | 1.49 (1.26-1.75) | 24 398 | 4.9 | 4 632 | 8.4 | 1.99 (1.86-2.12) |
| **By family history of psychiatric disorder** |  |  |  |  |  |  |  |  |  |  |
| No | 26 406 | 4.2 | 18 201 | 6.4 | 1.55 (1.51-1.58) | 218 922 | 3.8 | 29 369 | 6.3 | 1.66 (1.64-1.68) |
| Yes | 11 961 | 6.0 | 7 592 | 9.2 | 1.56 (1.51-1.61) | 70 515 | 5.0 | 12 110 | 9.9 | 1.89 (1.84-1.94) |

CVD: cardiovascular disease.

^a^Cox regression models, stratified by family identifier for sibling comparison or matching identifier (birth year and sex) for population comparison, adjusting for sex, birth year, educational level, individualized family income, cohabitation status, history of somatic disease and family history of psychiatric disorder. Time since index date was used as underlying time scale.

**1d. Crude incidence rates (IRs) and hazard ratios (HRs) with 95% confidence intervals (CIs) for incident psychiatric disorders among CVD patients compared with their full siblings or matched population controls, by time of follow-up (<1 or >=1 year from CVD diagnosis)**

|  | Sibling comparison | | | Population comparison | | |
| --- | --- | --- | --- | --- | --- | --- |
|  | No. of cases | IR (per 1000 person years) | HR (95% CI)^a^ | No. of cases | IR (per 1000 person years) | HR (95% CI)^a^ |
| **<1 year follow-up** |  |  |  |  |  |  |
| Full siblings/matched population controls | 4 024 | 4.6 | 1.0 | 33 625 | 4.1 | 1.0 |
| CVD patients_controlled for sex, birth year, educational level, individualized family income and cohabitation status | 5 695 | 12.6 | 2.76 (2.64-2.89) | 9 545 | 12.6 | 3.06 (2.99-3.14) |
| As above + history of somatic diseases |  |  | 2.74 (2.62-2.87) |  |  | 3.02 (2.95-3.09) |
| As above + family history of psychiatric disorder |  |  | - |  |  | 3.00 (2.93-3.07) |
| **>=1 year follow-up** |  |  |  |  |  |  |
| Full siblings/ matched population controls | 36 021 | 4,8 | 1.0 | 271 900 | 4.3 | 1.0 |
| CVD patients_controlled for sex, birth year, educational level, individualized family income and cohabitation status | 23 955 | 6.9 | 1.46 (1.43-1.49) | 38 414 | 6.9 | 1.60 (1.58-1.61) |
| As above + history of somatic diseases |  |  | 1.45 (1.42-1.48) |  |  | 1.56 (1.54-1.57) |
| As above + family history of psychiatric disorder |  |  | - |  |  | 1.54 (1.53-1.56) |

CVD: cardiovascular disease.

^a^Cox regression models, stratified by family identifier for sibling comparison or matching identifier (birth year and sex) for population comparison. Time since index date was used as underlying time scale.

**1e. Crude incidence rates (IRs) of different types of psychiatric disorder among CVD patients, their full siblings, and matched population controls, by time of follow-up (<1 or >=1 year from CVD diagnosis)**

|  | Sibling comparison | | | | Population comparison | | | |
| --- | --- | --- | --- | --- | --- | --- | --- | --- |
|  | Unaffected full siblings | | CVD patients | | Matched population controls | | CVD patients | |
|  | No. of cases | IR (per 1000 person years) | No. of cases | IR (per 1000 person years) | No. of cases | IR (per 1000 person years) | No. of cases | IR (per 1000 person years) |
| **<1 year follow up** |  |  |  |  |  |  |  |  |
| Non-affective psychotic disorders | 111 | 0.1 | 125 | 0.3 | 879 | 0.1 | 227 | 0.3 |
| Affective psychotic disorders | 132 | 0.2 | 131 | 0.3 | 1 177 | 0.1 | 234 | 0.3 |
| Alcohol or drug misuse | 845 | 1.0 | 1 031 | 2.3 | 8 848 | 1.1 | 1 786 | 2.4 |
| Mood disorders, excluding those with psychotic symptoms | 956 | 1.1 | 1 353 | 3.0 | 8 660 | 1.0 | 2 341 | 3.1 |
| Anxiety and stress related disorders | 1 649 | 1.9 | 2 620 | 5.8 | 11 954 | 1.4 | 4 263 | 5.6 |
| Eating disorders | 34 | 0.0 | 75 | 0.2 | 268 | 0.0 | 96 | 0.1 |
| Personality disorders | 48 | 0.1 | 47 | 0.1 | 294 | 0.0 | 84 | 0.1 |
| **>=1 year follow up** |  |  |  |  |  |  |  |  |
| Non-affective psychotic disorders | 1 048 | 0.1 | 519 | 0.2 | 6 109 | 0.1 | 919 | 0.2 |
| Affective psychotic disorders | 1 155 | 0.2 | 632 | 0.2 | 8 226 | 0.1 | 1 023 | 0.2 |
| Alcohol or drug misuse | 7 906 | 1.1 | 5 516 | 1.6 | 66 544 | 1.0 | 8 959 | 1.6 |
| Mood disorders, excluding those with psychotic symptoms | 9 079 | 1.2 | 6 072 | 1.7 | 70 301 | 1.1 | 9 852 | 1.8 |
| Anxiety and stress related disorders | 14 301 | 1.9 | 9 533 | 2.7 | 108 054 | 1.7 | 14 999 | 2.7 |
| Eating disorders | 269 | 0.0 | 188 | 0.1 | 1 654 | 0.0 | 264 | 0.1 |
| Personality disorders | 378 | 0.1 | 261 | 0.1 | 2 235 | 0.0 | 415 | 0.1 |

CVD: cardiovascular disease.

**1f. Crude incidence rates (IRs) for psychiatric disorders among different groups of CVD patients, their full siblings and matched population controls, by time of follow-up (<1 or >=1 year from CVD diagnosis)^a^**

|  | Sibling comparison | | Population comparison | |
| --- | --- | --- | --- | --- |
|  | No. of cases | IR (per 1000 person years) | No. of cases | IR (per 1000 person years) |
| **<1 year follow-up** |  |  |  |  |
| Unaffected siblings/individuals | 4 024 | 4.6 | 33 625 | 4.1 |
| 1. Ischemic heart disease | 1 043 | 9.7 | 1 751 | 9.4 |
| 2. Cerebrovascular disease | 1 039 | 17.0 | 1 793 | 16.7 |
| 3. Emboli and thrombosis | 301 | 14.1 | 488 | 13.8 |
| 4. Hypertensive disease | 1,124 | 13.5 | 1 941 | 14.0 |
| 5. Heart failure, all | 177 | 15.6 | 324 | 15.1 |
| 6. Arrhythmia/conduction disorder | 1 445 | 12.7 | 2 332 | 12.5 |
| **>=1 year follow-up** |  |  |  |  |
| Unaffected siblings/individuals | 36 021 | 4.8 | 271 900 | 4.3 |
| 1. Ischemic heart disease | 4 253 | 4.8 | 7 024 | 4.7 |
| 2. Cerebrovascular disease | 3 354 | 7.5 | 5 539 | 7.5 |
| 3. Emboli and thrombosis | 1 143 | 7.9 | 1 819 | 8.1 |
| 4. Hypertensive disease | 4 294 | 7.5 | 7 000 | 7.6 |
| 5. Heart failure, all | 614 | 8.4 | 1 080 | 8.2 |
| 6. Arrhythmia/conduction disorder | 6 842 | 7.7 | 10 668 | 7.7 |

CVD: cardiovascular disease.

^a^ We identified all cardiovascular diagnoses during follow-up and considered CVD comorbidity as a time-varying variable by grouping the person-time according to each diagnosis.

**1g. Crude incidence rates (IRs) and hazard ratios (HRs) with 95% confidence intervals (CIs) for psychiatric disorders among CVD patients compared with their full siblings or matched population controls, excluding CVD patients medicated with psychotropic drugs, by time of follow-up (<1 or >=1 year from CVD diagnosis)^a^**

|  | Sibling comparison**^§^** | | | Population comparison**^§^** | | |
| --- | --- | --- | --- | --- | --- | --- |
|  | No. of cases | IR (per 1000 person years) | HR (95% CI)^b^ | No. of cases | IR (per 1000 person years) | HR (95% CI)^b^ |
| **<1 year follow up** |  |  |  |  |  |  |
| Full siblings/matched population controls | 12 439 | 35.4 | 1.0 | 129 332 | 36.3 | 1.0 |
| CVD patients_controlled for sex, birth year, educational level, individualized family income and cohabitation status | 22 609 | 122.2 | 3.73 (3.62-3.83) | 40 008 | 130.5 | 3.62 (3.57-3.66) |
| As above + history of somatic diseases |  |  | 3.70 (3.60-3.81) |  |  | 3.60 (3.56-3.65) |
| As above + family history of psychiatric disorder |  |  | - |  |  | 3.61 (3.56-3.65) |
| **>=1 year follow up** |  |  |  |  |  |  |
| Full siblings/ matched population controls | 45 253 | 32.2 | 1.0 | 466 103 | 33.8 | 1.0 |
| CVD patients_controlled for sex, birth year, educational level, individualized family income and cohabitation status | 31 508 | 43.6 | 1.41 (1.39-1.44) | 55 074 | 47.4 | 1.39 (1.37-1.40) |
| As above + history of somatic diseases |  |  | 1.40 (1.38-1.43) |  |  | 1.39 (1.38-1.41) |
| As above + family history of psychiatric disorder |  |  | - |  |  | 1.40 (1.38-1.41) |
|  |  |  |  |  |  |  |
| **For incident eating disorder** |  |  |  |  |  |  |
| <1 year follow up |  |  |  |  |  |  |
| Full siblings/matched population controls | 26 | 0.07 | 1.0 | 188 | 0.05 | 1.0 |
| CVD patients_controlled for sex, birth year, educational level, individualized family income and cohabitation status | 49 | 0.26 | 6.50 (2.93-14.43) | 61 | 0.20 | 4.01 (2.96-5.43) |
| As above + history of somatic diseases |  |  | 6.61 (2.95-14.80) |  |  | 3.98 (2.93-5.39) |
| As above + family history of psychiatric disorder |  |  | - |  |  | 4.10 (3.02-5.57) |
|  |  |  |  |  |  |  |
| >=1 year follow up |  |  |  |  |  |  |
| Full siblings/ matched population controls | 88 | 0.06 | 1.0 | 550 | 0.04 | 1.0 |
| CVD patients _controlled for sex, birth year, educational level, individualized family income and cohabitation status | 60 | 0.08 | 1.52 (0.93-2.48) | 85 | 0.07 | 1.67 (1.32-2.11) |
| As above + history of somatic diseases |  |  | 1.51 (0.93-2.47) |  |  | 1.66 (1.31-2.09) |
| As above + family history of psychiatric disorder |  |  | - |  |  | 1.65 (1.31-2.09) |

CVD: cardiovascular disease.

^a^ CVD patients diagnosed during 2006-2016 were included in this analysis due to the availability of data on prescribed drug. In sibling comparison, 27.8% CVD patients and 23.5% siblings were excuded due to prior medicaiton of psychotropic drugs before index date. In population comparison, 30.6% CVD patients and 25.0% population controls were excluded due to prior medicated with psychotropic drugs before index date.

^b^ Cox regression models, stratified by family identifier for sibling comparison or matching identifier (birth year and sex) for population comparison. Time since index date was used as underlying time scale. Definition of psychiatric disorder included hospital visits as well as use of psychotropic drugs during follow-up.


**1h. Crude incidence rates (IRs) and hazard ratios (HRs) with 95% confidence intervals (CIs) for incident psychiatric disorder among CVD patients compared with their full siblings or matched population controls, restricting study period to 2001-2016 and excluding individuals with a history of alcoholic cirrhosis of liver or COPD, by time of follow-up (<1 or >=1 year from CVD diagnosis).**

|  | Sibling comparison§ | | | Population comparison§ | | |
| --- | --- | --- | --- | --- | --- | --- |
|  | No. of cases | IR (per 1000 person years) | HR (95% CI)* | No. of cases | IR (per 1000 person years) | HR (95% CI)* |
| <1 year follow up |  |  |  |  |  |  |
| Full siblings/matched population controls | 3,465 | 5.30 | 1.0 | 28,385 | 4.40 | 1.0 |
| CVD patients_controlled for sex, birth year, educational level, individualized family income and cohabitation status | 4,781 | 13.79 | 2.68 (2.55-2.81) | 8,021 | 13.68 | 3.08 (3.00-3.16) |
| As above + history of somatic diseases |  |  | 2.66 (2.53-2.79) |  |  | 3.03 (2.95-3.11) |
| As above + family history of psychiatric disorder |  |  | - |  |  | 3.01 (2.94-3.09) |
|  |  |  |  |  |  |  |
| >=1 year follow up |  |  |  |  |  |  |
| Full siblings/ matched population controls | 20,022 | 5.10 | 1.0 | 153,424 | 4.24 | 1.0 |
| CVD patients _controlled for sex, birth year, educational level, individualized family income and cohabitation status | 12,745 | 6.96 | 1.37 (1.34-1.41) | 20,596 | 6.99 | 1.60 (1.57-1.62) |
| As above + history of somatic diseases |  |  | 1.36 (1.33-1.40) |  |  | 1.55 (1.52-1.57) |
| As above + family history of psychiatric disorder |  |  | - |  |  | 1.54 (1.52-1.56) |

COPD, chronic obstructive pulmonary disease;

§ In sibling comparison, 1.14% exposed patients and 0.55% siblings were excluded due to a history of alcoholic cirrhosis or COPD before index date. In population comparison, 1.44% exposed patients and 1.01% population controls were excluded due to having a history of alcoholic cirrhosis or COPD before index date.

*Cox regression models, stratified by family identifier for sibling comparison or matching identifier (birth year and sex) for population comparison. Time since index date was used as underlying time scale. Definition of psychiatric disorder included hospital visits as well as use of psychotropic drugs during follow-up.
