# Supplementary material for: Cardiovascular disease and subsequent risk of psychiatric disorders: a nationwide sibling-controlled study
Source: eLife. 2022 Oct 21;11:e80143. doi: 10.7554/eLife.80143 (PMC9718522; doi:10.7554/eLife.80143)
Supplement: Supplementary file 2. — CVD: cardiovascular disease. *67,745 families had more than one sibling affected by CVD. [file elife-80143-supp2.docx]

Supplementary File 2 - Study design

CVD: cardiovascular disease.

* 67 745 families had more than one sibling affected by CVD.
